# Magnetic resonance imaging features of tumor and lymph node to predict clinical outcome in node-positive cervical cancer: a retrospective analysis

**DOI:** 10.1186/s13014-020-01502-w

**Published:** 2020-04-20

**Authors:** Shin-Hyung Park, Myong Hun Hahm, Bong Kyung Bae, Gun Oh Chong, Shin Young Jeong, Sungdae Na, Sungmoon Jeong, Jae-Chul Kim

**Affiliations:** 1 Department of Radiation Oncology, School of Medicine, Kyungpook National University, Daegu, Republic of Korea; 2 Department of Radiology, School of Medicine, Kyungpook National University, Daegu, Korea Republic of Korea; 3 Department of Obstetrics and Gynecology, School of Medicine, Kyungpook National University, Daegu, Republic of Korea; 4 Department of Obstetrics and Gynecology, Kyungpook National University Chilgok Hospital, Daegu, Republic of Korea; 5 Molecular Diagnostics and Imaging Center, School of Medicine, Kyungpook National University, Daegu, Republic of Korea; 6 Department of Nuclear Medicine, School of Medicine, Kyungpook National University, Daegu, Republic of Korea; 7 Department of Biomedical Engineering Center, Kyungpook National University Hospital, Daegu, Republic of Korea; 8 Bio-Medical Research Institute, School of Medicine, Kyungpook National University, Daegu, Republic of Korea; 9 Center for Artificial Intelligence in Medicine, Kyungpook National University Hospital, Daegu, Republic of Korea

**Keywords:** Cervical cancer, Lymph node, Chemoradiotherapy, Predictive modeling, Texture analysis, Radiomics

## Abstract

**Background:**

Current chemoradiation regimens for locally advanced cervical cancer are fairly uniform despite a profound diversity of treatment response and recurrence patterns. The wide range of treatment responses and prognoses to standardized concurrent chemoradiation highlights the need for a reliable tool to predict treatment outcomes. We investigated pretreatment magnetic resonance (MR) imaging features of primary tumor and involved lymph node for predicting clinical outcome in cervical cancer patients.

**Methods:**

We included 93 node-positive cervical cancer patients treated with definitive chemoradiotherapy at our institution between 2006 and 2017. The median follow-up period was 38 months (range, 5–128). Primary tumor and involved lymph node were manually segmented on axial gadolinium-enhanced T1-weighted images as well as T2-weighted images and saved as 3-dimensional regions of interest (ROI). After the segmentation, imaging features related to histogram, shape, and texture were extracted from each ROI. Using these features, random survival forest (RSF) models were built to predict local control (LC), regional control (RC), distant metastasis-free survival (DMFS), and overall survival (OS) in the training dataset (*n* = 62). The generated models were then tested in the validation dataset (*n* = 31).

**Results:**

For predicting LC, models generated from primary tumor imaging features showed better predictive performance (C-index, 0.72) than those from lymph node features (C-index, 0.62). In contrast, models from lymph nodes showed superior performance for predicting RC, DMFS, and OS compared to models of the primary tumor. According to the 3-year time-dependent receiver operating characteristic analysis of LC, RC, DMFS, and OS prediction, the respective area under the curve values for the predicted risk of the models generated from the training dataset were 0.634, 0.796, 0.733, and 0.749 in the validation dataset.

**Conclusions:**

Our results suggest that tumor and lymph node imaging features may play complementary roles for predicting clinical outcomes in node-positive cervical cancer.

## Background

Medical imaging has profound clinical importance for diagnosis, staging, treatment, and predicting prognosis in cancer patients. In current practice, the majority of clinical decisions are based on a limited number of radiologic features that can be readily processed by the unaided radiologist’s eye. However, tumor imaging may contain more information than can be visually assessed. For this reason, recent studies have suggested that quantitative imaging features of the tumor mass, including shape and texture, may also have prognostic importance for predicting patient outcomes in various cancer sites [1–3].

The standard treatment for locally advanced cervical cancer is cisplatin-based concurrent chemoradiation. Despite the use of combined-modality treatment with external beam radiotherapy, intracavitary brachytherapy, and chemotherapy, approximately 30% of these patients experience progression and recurrence [4–6]. In the updated results from the Radiation Therapy Oncology Group Trial 90–01 with a median follow-up of 6.6 years for 228 survivors, the 5-year cumulative disease progression rate of stage IIB-IVA patients who were treated with cisplatin-based concurrent chemoradiation was reported as 32% [4]. Nevertheless, current chemoradiation regimens for locally advanced cervical cancer remain fairly uniform despite a profound diversity of treatment response and recurrence patterns. The wide range of treatment responsiveness and prognoses, despite the administration of standard concurrent chemoradiation, highlights the need for a reliable tool to predict treatment outcomes.

Previous studies have shown that imaging features extracted from magnetic resonance (MR) imaging can provide information on the likelihood of tumor characteristics in cervical cancer [7–9]. However, these studies have primarily focused on investigating the predictive performance of imaging features in terms of classification of lymph node metastasis or molecular characteristics, rather than clinical outcomes such as recurrence, distant metastasis, and overall survival. Furthermore, to our knowledge, no study has assessed the predictive performance of imaging features obtained both from primary tumors and involved lymph nodes in cervical cancer. Therefore, we investigated the pretreatment MR imaging features of primary tumor and involved lymph node for predicting local control (LC), regional control (RC), distant metastasis-free survival (DMFS), and overall survival (OS) in cervical cancer patients.

## Methods

### Patient, tumor, and treatment characteristics

We retrospectively reviewed the medical records of 121 consecutive cervical cancer patients who were treated with definitive concurrent chemoradiotherapy at our institution between 2006 and 2017. Of these 121 patients, 28 patients were not evaluated with pretreatment MR imaging. Thus, a final total of 93 patients were included in the analysis. The institutional review board approved this study and provided a waiver of consent (2017–06-032). A positive lymph node was defined as having a maximum short axis diameter of ≥8 mm according to pretreatment MR imaging [10, 11].

The characteristics of the analyzed patients are listed in Table 1. The median follow-up period was 38 months (range, 5–128). The median age of patients at diagnosis was 53 years (range, 23–82). Of the 93 included patients, 86 (92.5%) had squamous cell carcinoma, 5 (5.4%) had adenocarcinoma, and 2 (2.2%) had adenosquamous carcinoma. The International Federation of Gynecology and Obstetrics (FIGO) stage was IIB in 77 patients (82.8%), IIIA in 5 (5.4%), and IIIB in 11 (11.8%) patients. Seventy-seven patients (82.8%) had pelvic node involvement only, and 16 patients (17.2%) had both pelvic and para-aortic node involvement.

**Table 1:** Patient and tumor characteristics

	n (%)
	Total *n* = 93
Age (years)	
Median (range)	53 (23–82)
Pathology	
Squamous cell carcinoma	86 (92.5%)
Adenocarcinoma	5 (5.4%)
Adenosquamous carcinoma	2 (2.1%)
FIGO stage*	
IIB	77 (82.8%)
IIIA	5 (5.4%)
IIIB	11 (11.8%)
Primary tumor size (mm)	
< 50	45 (48.4%)
≥ 50	48 (51.6%)
Extent of lymph node involvement	
Pelvic only	77 (82.8%)
Pelvic + para-aortic	16 (17.2%)

*The 2009 International Federation of Gynecology and Obstetrics (FIGO) staging system

All patients were treated with a combination of external beam radiotherapy (EBRT) followed by high-dose-rate (HDR) intracavitary brachytherapy (ICR) with curative intent. EBRT was delivered to the whole pelvis using a 3-dimensional conformal radiation therapy (3D-CRT) 4-field box technique (1.8 Gy daily fractions, 5 times per week, for a total dose of 45 Gy). Extended-field radiotherapy, including pelvis and para-aortic nodal area, was administered to the patients with para-aortic nodal involvement. HDR ICR was initiated after delivery of an EBRT dose of 39.6 Gy. An additional 5.4 Gy was administered with a midline block. An additional parametrial boost of 10 Gy in 5 fractions was given to patients with parametrial involvement. ICR was delivered twice per week in 5 fractions with a fractional dose of 6 Gy at point A. Weekly cisplatin at a dose of 40 mg/m^2^ was administered during radiotherapy. The first course of cisplatin was administered on day 1 of radiotherapy.

### MR acquisition

MR images were obtained with one 3.0 T and two 1.5 T MR imaging units (Discovery MR750, GE Healthcare; Magnetom Avanto, Siemens Healthcare; Signa Excite, GE Healthcare), with a pelvic array coil for the pelvic scans. We obtained the same MR imaging sequences for all patients, including axial and sagittal T2-weighted fast spin-echo (FSE), axial T1-weighted FSE, and axial and sagittal T1-weighted FSE with fat saturation after administration of gadodiamide (Omniscan; Nycomed Imaging) at a dose of 0.1 mmol/kg body weight. The magnetic resonance protocol used the following parameters: axial T2-weighted images (repetition time (TR)/echo time (TE), 3500–4500/90–110; slice thickness, 5 mm, no gap; field of view, 22 × 22 cm to 26 × 26 cm; matrix, 320 × 224, 384 × 256), sagittal T2-weighted images (TR/TE, 4000–6000/90–110; slice thickness, 5 mm, no gap; field of view, 24 × 24 cm; matrix, 384 × 256, 416 × 256), axial T1-weighted images (TR/TE, 700–800/minimum; slice thickness, 5 mm, no gap; field of view, 22 × 22 cm to 26 × 26 cm; matrix, 320 × 256, 384 × 224).

### Segmentation

**Fig. 1:**
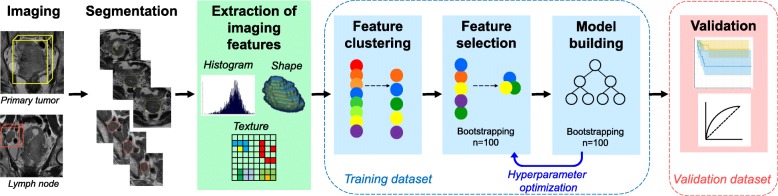
Illustrations of the key steps in the imaging feature analysis process. First, primary cervical tumor and involved lymph node were segmented. Second, imaging features were extracted from each region of interest. Third, random survival forest models were built to predict survival outcomes, after the feature clustering and selection process in the training dataset. Fourth, the generated models were tested in the validation dataset

The key steps of the imaging feature analysis process are illustrated in Fig. 1. Patient-sensitive information was anonymized before image segmentation. Primary tumor tissue and involved lymph nodes were manually segmented on the axial gadolinium-enhanced T1-weighted images (T1WI) and T2-weighted images (T2WI) by 2 radiation oncologists (S.H and B.B) using the annotation tool of the m:Studio Research Platform [12]. In case of multiple lymph node involvement, the largest lymph node was selected for segmentation. Segmented contours of tumor and lymph node were subsequently reviewed and revised by 1 radiologist (M.H). Each 3-dimensional region of interest (ROI) was saved as voxels.

### Feature extraction, clustering, and selection

After segmentation, 86 imaging features were extracted from each ROI segmented on enhanced T1WI and T2WI. These included (i) 12 first-order features, (ii) 6 grey-level co-occurrence matrix (GLCM) features, (iii) 11 grey-level run-length matrix (GLRLM), (iv) 3 neighborhood grey-level difference matrix (NGLDM), and (v) 11 grey-level zone length matrix (GLZLM). All matrices were calculated in a 3-dimensional manner using LIFEx software (Additional file 1) [13]. The number of grey levels was set at 64 (ROIs were discretized using 64 levels before feature extraction). To normalize the image intensities from different MR units, resampling was conducted as a relative value (between the minimum and maximum values in the ROI) [14].

After extraction, features were clustered to reduce dimensionality and to avoid multicollinearity. Spearman’s correlation analysis was performed, and highly correlated features (Spearman’s coefficient (SC) > 0.90) were clustered using hierarchical clustering. The resultant clusters were represented by a new feature calculated by averaging all features within a cluster. Negatively correlated features were inverted before being averaged.

The patient cohort was randomly divided into 2 independent groups for the training (62 patients) and validation (31 patients) datasets. After clustering, the feature set of the training dataset was used to identify the most relevant features using the Ridge, Lasso, and Elastic-net regularization algorithms. To improve the stability of the feature selecting process, feature selection was repeated *n* = 100 times using n bootstrap samples of the training dataset. The top 10 ranked features were selected from each bootstrap sample, and the selected features were aggregated over the bootstraps. We performed rank aggregation, the process of combining information from several ranked lists into a single, more stable list. The simple ensemble method was used to aggregate feature ranks [15].

### Model building and validation

We used the random survival forest (RSF) method to generate prediction models. RSF is an ensemble tree method for the analysis of right-censored survival data [16] that extends upon Breiman’s random forest approach [17]. For hyper-parameter optimization, grid search and cross validation was performed. Model performance was assessed in the validation cohort using the concordance index (C-index). Harrell’s C-index is a generalization of the area under the curve (AUC) for continuous time-to-event survival data [18]. C-index = 0.5 describes a random prediction, whereas a perfectly predicting model would have a C-index of 1.0. The R packages “randomForestSRC” in version 2.9.0 and “caret” in version 6.0–83 were used. Because the C-index approach may be problematic in situations with a fixed prediction time point [19], we also assessed t-year risk of an event using time-dependent area under the receiver operating curve analysis [20, 21]. To evaluate the predictive performance of the built RSF models, we divided the patients into low- and high-risk groups according to predicted risk. The optimal cutoff value was calculated based on our dataset using the “cutp” function of the R package “survMisc” in version 0.5.5. In image processing and feature calculation, we followed guidelines of the Image Biomarkers Standardization Initiative [22].

### Statistical analysis

Three-year actuarial rates of LC, RC, DMFS, and OS were calculated using the Kaplan–Meier method, and comparisons among groups were conducted using 2-sided log-rank tests. These endpoints were reached at the first observation of a defined event, and all events were calculated from the start of definitive chemoradiotherapy. For LC, the first event could present as persistent disease, or recurrence in the cervix or an adjacent pelvic organ. For RC, the first event was defined as either a persistent node or recurrence in the pelvic or para-aortic area. For DMFS, the events included recurrence at any other site or death from any cause. For OS, the event was death from any cause. All statistical analyses were performed using R project (version 3.5.3).

## Results

**Fig. 2:**
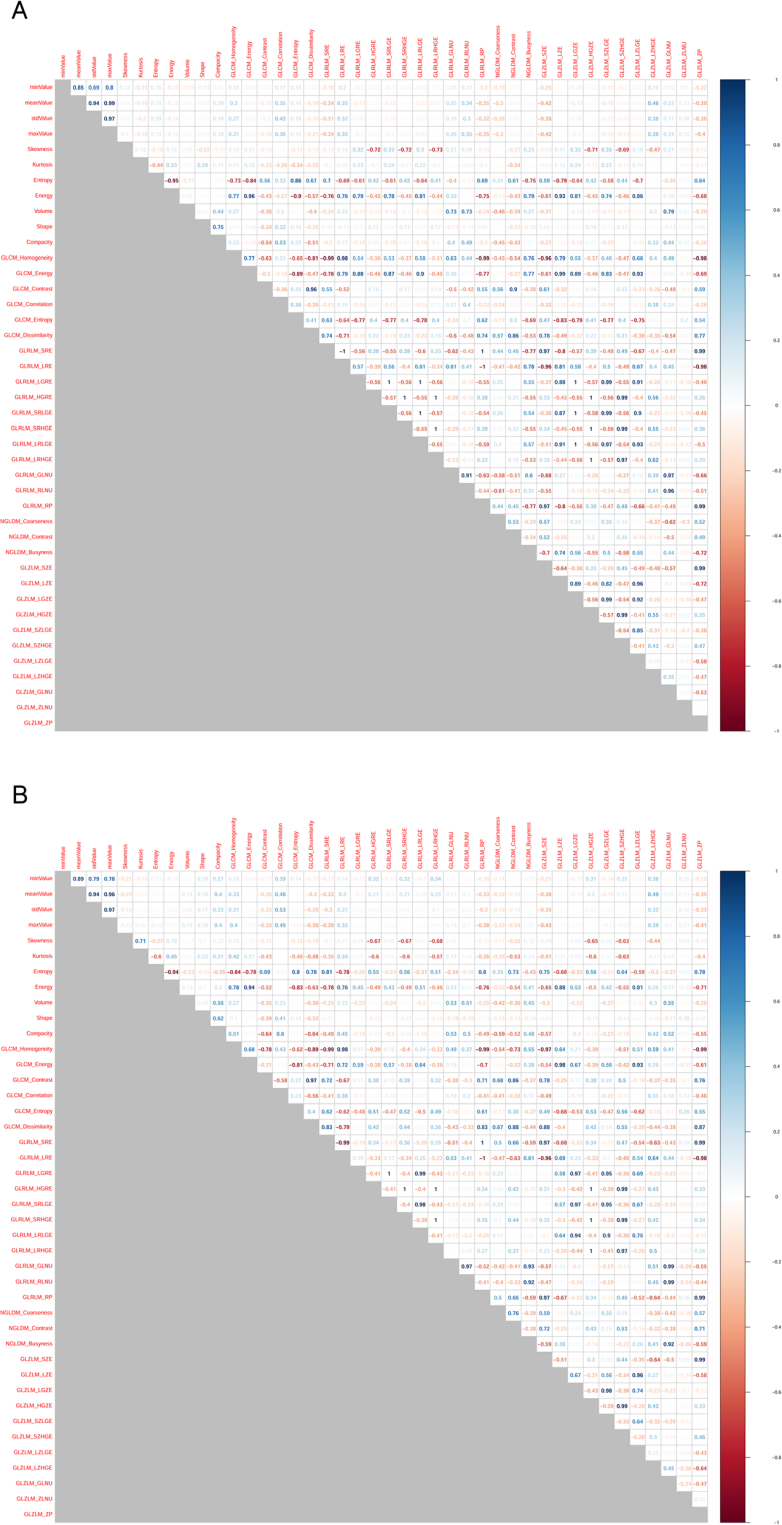
Spearman’s correlation coefficients for all imaging features of primary cervical tumors on the axial gadolinium-enhanced T1-weighted (**a**) and T2-weighted images (**b**). Highly positive correlation coefficients are presented in blue, whereas highly negative correlation coefficients are in red

**Fig. 3:**
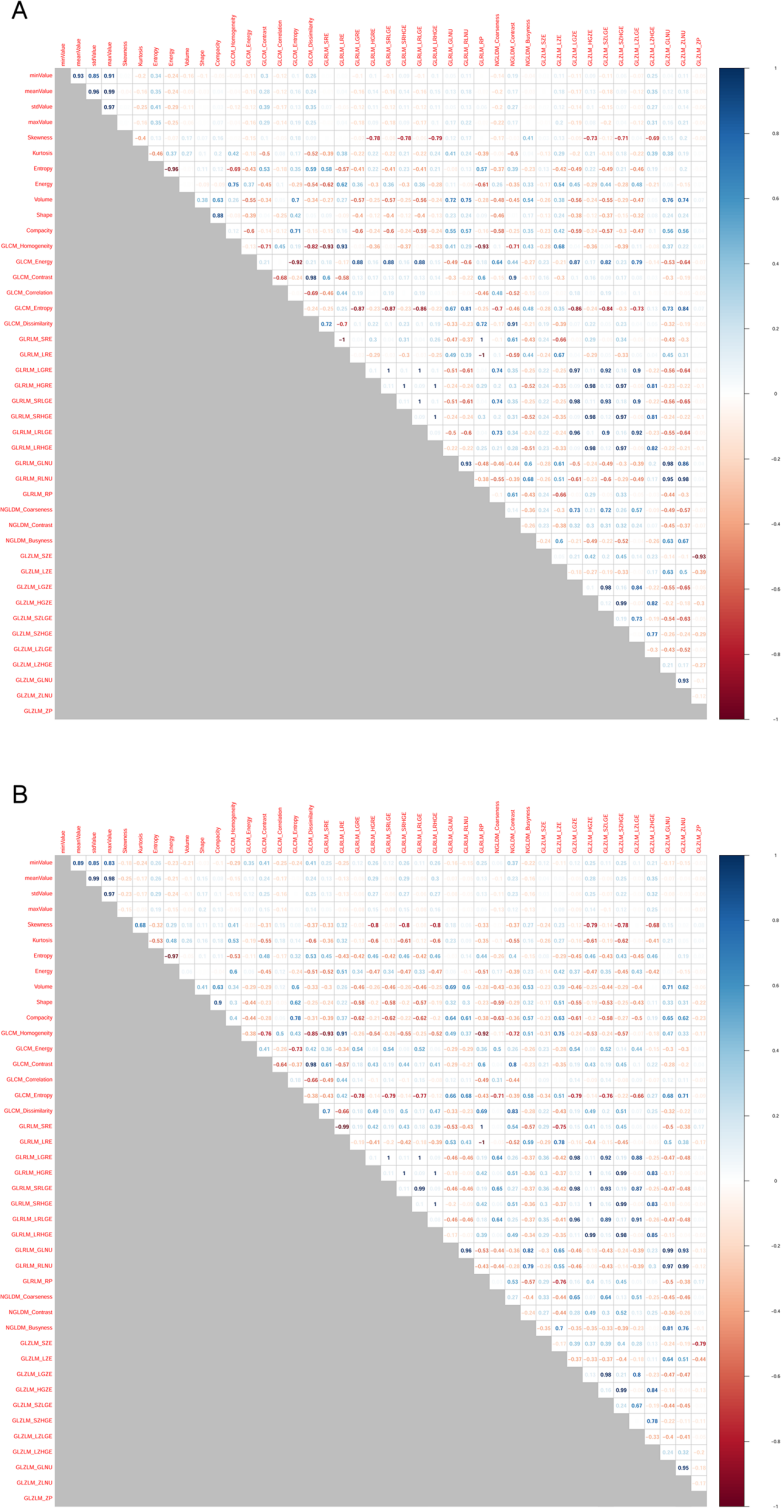
Spearman’s correlation coefficients for all imaging features of involved lymph nodes on the axial gadolinium-enhanced T1-weighted (**a**) and T2-weighted images (**b**). Highly positive correlation coefficients are presented in blue, whereas highly negative correlation coefficients are in red

**Fig. 4:**
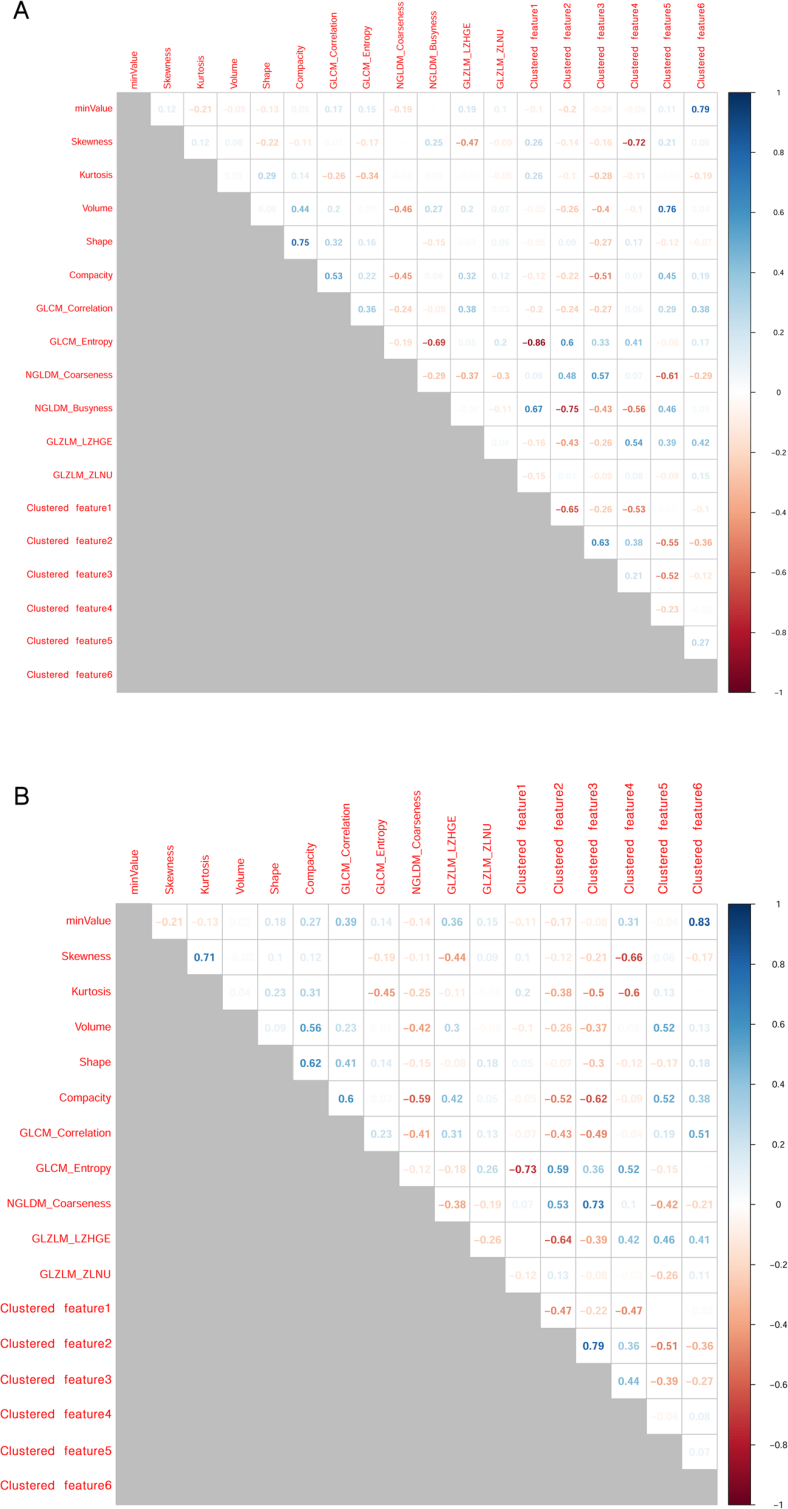
Clustered imaging features of primary cervical tumors on the axial gadolinium-enhanced T1-weighted (**a**) and T2-weighted images (**b**). No Spearman’s correlation coefficients > 0.90 were observed between clustered features

**Fig. 5:**
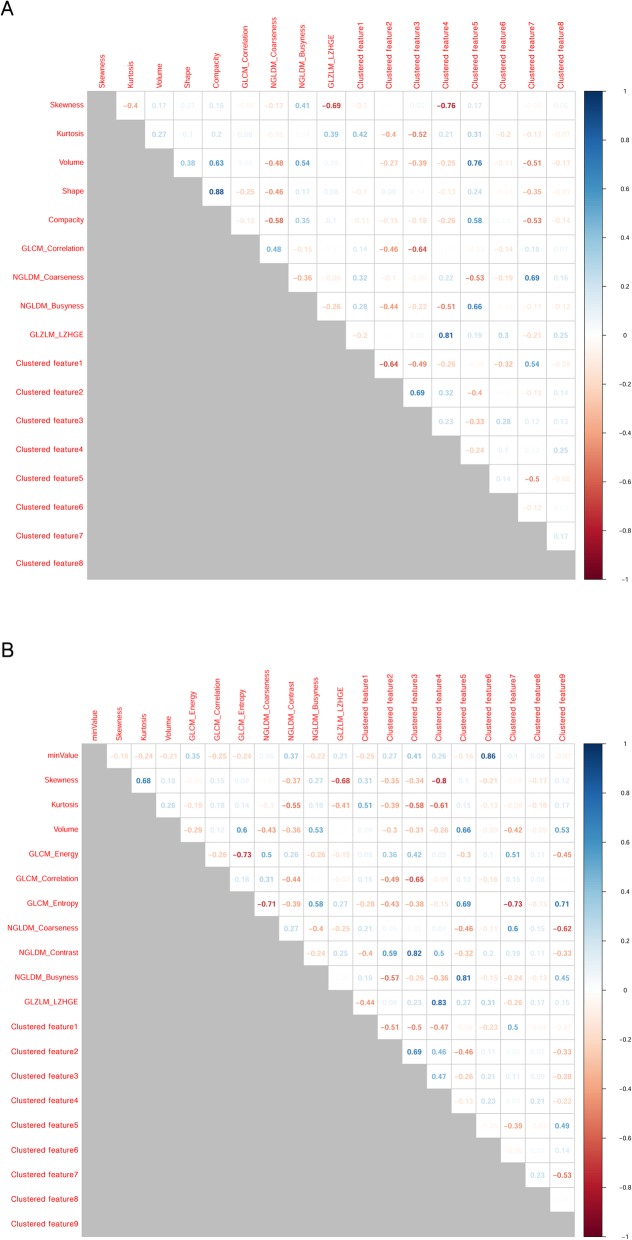
Clustered imaging features of involved lymph nodes on the axial gadolinium-enhanced T1-weighted (**a**) and T2-weighted images (**b**). No Spearman’s correlation coefficients > 0.90 were observed between clustered features

The SC values between features are summarized as a correlation matrix (Fig. 2-3). After feature clustering, no SC > 0.90 was observed between clustered features (Fig. 4-5). After the feature clustering process, 18 and 17 imaging features of primary cervical tumors, and 17 and 20 lymph node imaging features from the T1WI and T2WI, respectively, were used for modeling. Using the training dataset, RSF models predicting LC, RC, DMFS, and OS were built, and predictive performance was tested in the validation dataset. For predicting LC, the models using primary cervical tumor imaging features showed better predictive performance (C-index = 0.72 ± 0.08) (mean ± standard deviation) than the models from involved lymph nodes (C-index = 0.62 ± 0.12). In contrast, for predicting RC, DMFS, and OS, the models using imaging features of involved lymph nodes showed superior predictive performance (RC, C-index = 0.69 ± 0.07;DMFS, C-index = 0.66 ± 0.08; OS, C-index = 0.72 ± 0.11) compared to the models from primary cervical tumor features (RC, C-index = 0.65 ± 0.06; DMFS, C-index = 0.64 ± 0.07; OS, C-index = 0.69 ± 0.07).

**Fig. 6:**
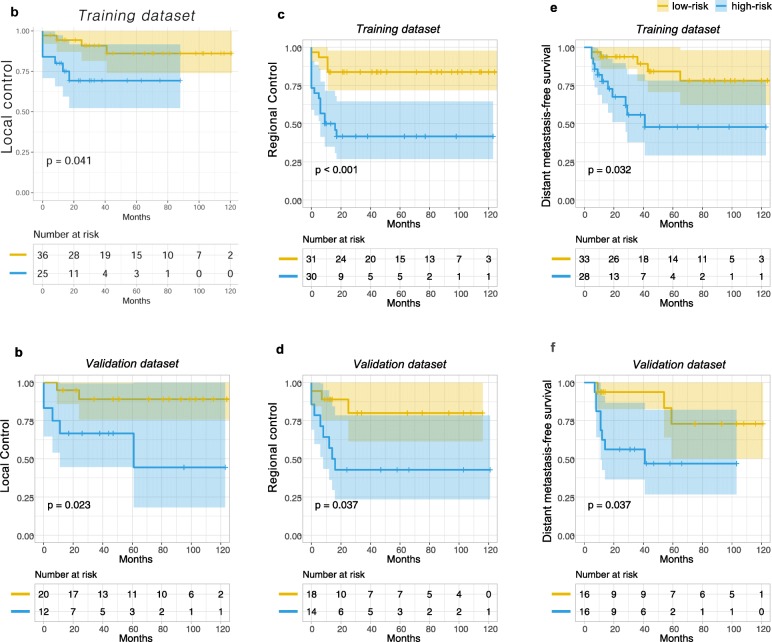
Kaplan–Meier curves of local control (**a**, **b**), regional control (**c**, **d**), and distant metastasis-free survival (**e**, **f**) for patients from the training dataset and validation dataset, respectively. Patients were stratified into a low-risk (yellow line) and a high-risk group (blue line) according to predicted risk from the random survival forest model based on pretreatment MRI features

**Fig. 7:**
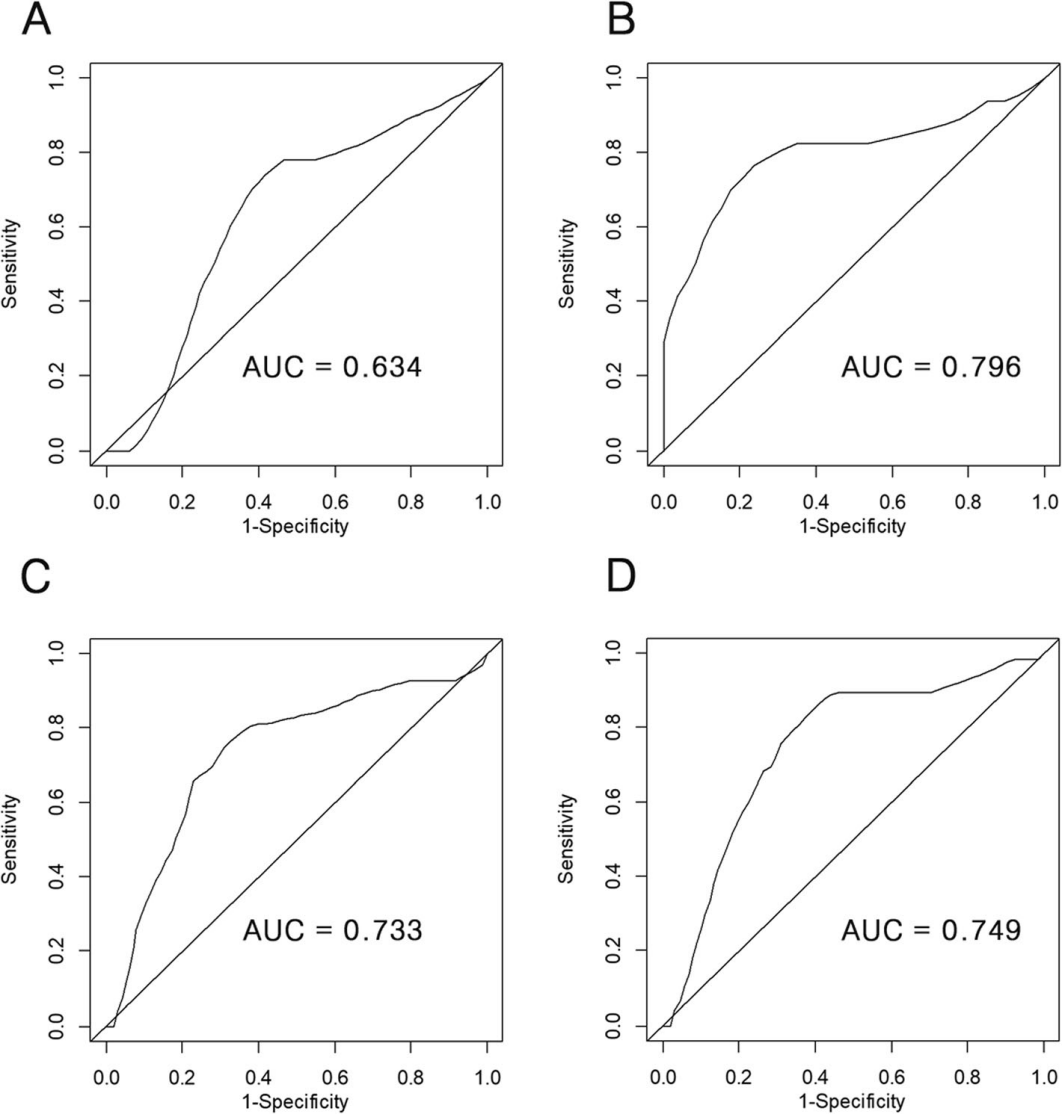
Time-dependent receiver operating characteristic curves for predicting 3-year clinical outcome. The predicted risk of the models showed area under the curve values of 0.634 for 3-year local control (**a**), 0.796 for 3-year regional control (**b**), 0.733 for 3-year distant metastasis-free survival (**c**), and 0.749 for 3-year overall survival (**d**), where patients were stratified according to the risk scores of a random survival forest model based on pretreatment MR imaging features

For each clinical endpoint, patients were divided into a low- and high-risk group based on the predicted risk of the models from the training dataset. Statistically significant differences between the low-risk and high-risk groups were observed for LC in both training and validation datasets when using primary cervical tumor imaging features (*P* = .041 and .023, respectively) (Table 2). In addition, statistically significant differences were demonstrated in both training and validation datasets between the low-risk and high-risk groups when using involved lymph node features for RC (*P* < .001 and .037, respectively) (Table 3) and DMFS (*P* = 0.032 and 0.037, respectively) (Table 4 and Figs. 6a, b, c, d, e and f). However, the models were not able to effectively stratify the patients in terms of OS into two groups with significantly different outcomes in the test dataset (Additional files 2 and 3). In the 3-year time-dependent receiver operating curve analysis of LC, RC, DMFS, and OS prediction, the predicted risk of the models showed AUC values of 0.634, 0.796, 0.733, and 0.749, respectively, in the validation dataset (Fig. 7).

**Table 2:** Univariate and multivariate analyses of potential prognostic factors for local control in the validation dataset. Statistically significant differences were found according to the imaging feature-based risk scores in the univariate and multivariate analyses

Variables		n	3-year LC (%)	*P* value		HR	95% CI
				univariate	multivariate		
Imaging feature-based LC risk score^a^	< 1.1	20	89.1	*0.023*^*b*^	*0.044*^*b*^	7.46	1.06–52.58
	≥1.1	12	66.7				
Age	< 50	15	60.6	0.160	0.161	5.50	0.50–59.52
	≥50	17	92.3				
Pathology	SCC	30	84.9	0.706	0.741	0.65	0.05–8.19
	Non-SCC	2	50.0				
FIGO stage^c^	IIB	25	81.9	0.213	1.00	< 0.01	0- ∞
	IIIA, IIIB	7	85.7				
Extent of nodal involvement	Pelvic only Pelvic + para-aortic	25	82.1	0.692	0.248	3.40	0.43-27.15
		7	85.7				
Primary tumor size (mm)	< 50	16	79.1	0.808	0.284	0.28	0.03–2.85
	≥50	16	87.5				

*Abbreviations*: *LC* local control, *HR* hazard ratio, *CI* confidence interval, *SCC* squamous cell carcinoma

^a^Imaging features of primary cervical tumor

^b^Significant *P* values

^c^The 2009 International Federation of Gynecology and Obstetrics (FIGO) staging system

**Table 3:** Univariate and multivariate analyses of potential prognostic factors for regional control in the validation dataset. Statistically significant differences were found according to the imaging feature-based risk scores in the univariate analysis

Variables		n	3-year RC (%)	*P* value		HR	95% CI
				univariate	multivariate		
Imaging feature-based RC risk score^a^	< 2.2	16	87.5	*0.025*^b^	0.058^b^	5.14	0.94–27.85
	≥2.2	16	42.9				
Age	< 50	15	49.5	0.155	0.542	0.67	0.18–2.45
	≥50	17	74.1				
Pathology	SCC	30	63.0	0.531	0.653	0.60	0.06–5.44
	Non-SCC	2	50.0				
FIGO stage^c^	IIB	25	58.8	0.743	0.856	0.85	0.16–4.62
	IIIA, IIIB	7	71.4				
Extent of nodal involvement	Pelvic only	25	63.1	0.516	0.394	2.01	0.40–10.02
	Pelvic + para-aortic	7	57.1				
Primary tumor size (mm)	< 50	16	50.8	0.377	0.172	0.43	0.11–1.64
	≥50	16	75.0				

*Abbreviations*: *RC* regional control, *HR* hazard ratio, *CI* confidence interval, *SCC* squamous cell carcinoma

^a^Imaging features of involved lymph nodes

^b^Significant *P* values

^c^The 2009 International Federation of Gynecology and Obstetrics (FIGO) staging system

**Table 4:** Univariate and multivariate analyses of potential prognostic factors for distant metastasis-free survival in the validation dataset. Statistically significant differences were found according to the imaging feature-based risk scores in the univariate and multivariate analyses

Variables		n	3-year DMFS (%)	*P* value		HR	95% CI
				univariate	multivariate		
Imaging feature-based DM risk score^a^	< 2.6	16	93.8	*0.037*^b^	*0.012*^b^	9.37	1.47–48.68
	≥2.6	16	56.2				
Age	< 50	15	71.5	0.707	0.678	1.30	0.37–4.53
	≥50	17	76.0				
Pathology	SCC	30	75.6	0.761	0.192	0.21	0.02–2.19
	Non-SCC	2	50.0				
FIGO stage^c^	IIB	25	73.4	0.609	0.589	0.62	0.11–3.57
	IIIA, IIIB	7	100.0				
Extent of nodal involvement	Pelvic only	25	74.4	0.690	0.201	2.73	0.59–12.76
	Pelvic + para-aortic	7	71.4				
Primary tumor size (mm)	< 50	16	67.3	0.129	0.073	0.21	0.04–1.16
	≥50	16	81.2				

*Abbreviations*: *RC* regional control, *HR* hazard ratio, *CI* confidence interval, *DM* distant metastasis, *SCC* squamous cell carcinoma

^a^Imaging features of involved lymph node

^b^Significant *P* values

^c^The 2009 International Federation of Gynecology and Obstetrics (FIGO) staging system

## Discussion

Using pretreatment MR imaging, we developed and validated RSF models for predicting LC, RC, DMFS, and OS in node-positive cervical cancer. The respective RSF models were built from imaging features both from primary tumors and involved lymph nodes. These models provided independent prognostic information beyond that of known clinicopathologic prognostic factors including FIGO stage, pathologic type, and the extent of lymph node involvement. Of note, the primary tumor imaging features demonstrated superior performance for predicting LC, while lymph node imaging features were superior at predicting RC, DMFS, and OS.

To date, a limited number of studies have been published concerning the relevance of imaging features with regard to cervical cancer disease characteristics or clinical outcomes. In most of these, the clinical endpoints were dichotomous variables and did not take into consideration of the time when that event occurred. For example, Becker et al. analyzed MR images of 23 patients with cervical cancer and found that imaging features extracted from apparent diffusion coefficient (ADC) maps were associated with histological differentiation and nodal stage [8]. Similarly, in an analysis of 34 patients with advanced cervical cancer by Meng et al., [23] T2WI- and ADC-based features were associated with disease recurrence. A study by Wu et al. of 189 cervical cancer patients reported that tumor imaging features on T2WI and ADC were highly predictive of lymph node metastasis (AUC and sensitivity of 0.842 and 100%, respectively, in the validation cohort) [7]. Sun et al. built random forest models with features extracted from both T1WI and T2WI to predict responses after neoadjuvant chemotherapy in cervical cancer patients. Unlike the studies cited above, our models predicted time-to-event survival outcomes rather than a dichotomized treatment response [34]. In this context, it is important to note that treatment responses often do not translate into improvements in overall survival [24].

Only limited data exist regarding the specific site of cervical cancer failure; local and regional failure have been reported as components of locoregional failure or pelvic failure. The recognition of specific patterns of failure may provide important information to guide which treatment needs to be modified from the radiation oncologists’ perspective. Our results suggest that imaging features extracted from the primary tumor may provide information for making decisions to escalate or de-escalate the radiation dose to the primary cervical tumor. For example, patients at high risk of local failure could benefit from an escalating dose to the uterine cervix, whereas patients at low risk of local failure could be considered for a de-escalating radiation dose to reduce the risk of radiation-induced toxicity. A number of significant late complications are associated with chemoradiation, including gastrointestinal, urologic, and gynecologic toxicities, particularly if intracavitary brachytherapy is added [5, 25]. For this reason, radiation dose need to be tailored according to the each patient’s given site-specific failure risk, not only to enhance failure-free survival but also to minimize treatment-related toxicity.

Another important finding of our study was that lymph node features demonstrated superior performance over primary tumor features for predicting RC and DMFS. While most previous work investigating imaging features has primarily focused on the primary tumor, we separately assessed involved lymph nodes, which could facilitate a more complete evaluation of disease status. For node-positive cervical cancer patients, it may be necessary to tailor doses according to the involved lymph nodes for personalized radiotherapy. Some investigators have reported that escalating radiotherapy dose to involved lymph node can improve RC [26–29]. Increased RC prediction accuracy using lymph node features may help select patients who require dose escalation to the involved lymph nodes. Additionally, the superior predictive performance of lymph node features for DMFS suggests that these features may contain information on metastatic potential of disease. This result is encouraging because the intensification of systemic therapy would be considered if we can select patients who are at high risk of distant metastasis.

Several modeling approaches can be applied to predict the risk of future events in terms of survival. The most widely used of these methods is the Cox-proportional hazards model. This model is flexible and simple, but it is difficult to apply in situations where the restrictive proportional hazards assumption is violated [30]. Moreover, in high-dimensional settings where the number of covariates far exceeds the number of observations, as in our study, standard survival analyses such as Cox-proportional hazard models might be inadequate. RSF is an ensemble method of building and splitting tree by maximizing the log-rank statistic in each node [16]. Ensemble predictions are given by averaging the cumulative hazard estimates in the terminal nodes of the trees. RSF has several advantages compared with regression-based methods. First, it is completely data-driven and thus independent of model-specific assumptions. Second, it seeks to generate a model that best explains the data and thus represents a suitable tool for exploratory analysis where prior information of the survival data is limited. Third, in cases of high-dimensional data, the limitations of univariate regression approaches, such as overfitting, unreliable estimation of regression coefficients, inflated standard errors or convergence problems, do not apply to RSF. Fourth, it is robust to outliers in the covariate space [31].

The common approach to evaluate the predictive performance of an RSF model is the Harrell’s C-index [18]. However, Blanche et al. demonstrated that C-index may not be the proper approach when predicting the risk of an event at a certain time point [19]. They noted that C-index assesses the order of event times rather than event status order at a given time point. Time-dependent ROC analysis does not have this problem [20, 21]. For this reason, we also performed time-dependent ROC analysis in both training and validation datasets to compare 3-year cumulative LC, RC, DMFS and OS between the low- and high-risk groups. Our results show that RSF models using imaging features could achieve an area under the 3-year time-dependent ROC of 0.634–0749 for predicting LC, RC, DMFS, and OS, indicating that imaging features could serve as biomarkers to discriminate low- and high-risk patients with moderate predictive accuracy. However, these levels of accuracy may not be sufficient to definitively predict prognosis for each patient’s prognosis by itself. This may suggest that other clinical, genomic, proteomic, and metabolomic factors could contribute to clinical outcomes, along with imaging features. Therefore, the use of multi-omics approaches could enable more accurate outcome predictions for each patient, paving the way to ‘personalized medicine’.

Our study has several limitations. First, its retrospective design and single-institution cohort may have a concealed selection bias. Although cross-validation and bootstrapping were used to compensate for such bias, there was a potential for overfitting. Second, we were not able to validate our models in external cohorts, although we did perform internal validation. Third, false-positive lymph nodes may have been included in the analysis. Fourth, substantial variations in MR image acquisition may have affected predictive performance. However, the good predictive values, regardless of MRI protocol, suggest that these imaging features may be robust in a variety of MR scanners or imaging protocols. Lastly, the imaging features in our study were not evaluated for ADC values in diffusion-weighted images (DWI) for prognosis prediction, in contrast to previous studies that primarily investigated ADC values in cervical cancer [32, 33]. This was because our patients were included to analyze long-term survival analysis; therefore, most pretreatment MR images were obtained before the role of DWI was established for patients with cervical cancer. Our future research will seek to augment these results using the imaging features extracted from DWI. Nevertheless, our current results provide valuable information about the predictive potential of pretreatment MR imaging and may provide baseline information useful for modifying the current uniform treatment for cervical cancer. We present the first quantitative analysis results separately evaluating the imaging features of primary tumors and lymph nodes in node-positive cervical cancer.

## Conclusions

We successfully developed and validated RSF models for predicting clinical outcomes using pretreatment MR imaging features. The models using primary tumor features demonstrated superior performance for predicting LC, while those using lymph node features were superior at predicting RC, DMFS, and OS. Our results indicate that tumor and lymph node imaging features may play complementary roles for predicting clinical outcomes in node-positive cervical cancer.

## Supplementary information

**Additional file 1.** Summary of imaging features.

**Additional file 2.** Univariate analysis of potential prognostic factors for overall survival in the validation dataset.

**Additional file 3.** Kaplan–Meier curves of overall survival for patients in the training dataset (A) and validation dataset (B) stratified into low- and a high-risk groups.

## Data Availability

The datasets generated and/or analyzed during the current study are not publicly available due to the privacy protection policy of personal medical information of our institution but are available from the corresponding author on reasonable request.

## Abbreviations

3D-CRT: 3-dimensional conformal radiation therapy
ADC: Apparent diffusion coefficient
AUC: Area under the curve
DMFS: Distant metastasis-free survival
DWI: Diffusion-weighted image
EBRT: External beam radiotherapy
FIGO: International Federation of Gynecology and Obstetrics
FSE: Fast spin-echo
GLCM: Grey-level co-occurrence matrix
GLRLM: Grey-level run-length matrix
GLZLM: Grey-level zone length matrix
HDR: High-dose rate
ICR: Intracavitary brachytherapy
LC: Local control
MR: Magnetic resonance
NGLDM: Neighborhood grey-level difference matrix
OS: Overall survival
RC: Regional control
ROI: Regions of interest
RSF: Random survival forest
SC: Spearman’s coefficient
T1WI: T1-weighted images
T2WI: T2-weighted images
TE: Echo time
TR: Repetition time
