# Supplementary material for: Magnetic resonance imaging features of tumor and lymph node to predict clinical outcome in node-positive cervical cancer: a retrospective analysis
Source: Radiat Oncol. 2020 Apr 20;15:86. doi: 10.1186/s13014-020-01502-w (PMC7171757; doi:10.1186/s13014-020-01502-w)
Supplement: Supplementary file 1 — Additional file 1. Summary of imaging features. [file 13014_2020_1502_MOESM1_ESM.pdf]

Supplementary Table 1. Summary of imaging features.

Imaging feature[1]	Formula
First-order features	
Mean (μ): average intensity values in an image	$\sum_i ip(i)$
Standard deviation: the spread or variation around the mean	$\sqrt{\sum_i (i-\mu)^2 p(i)}$
Minimum: minimal intensity values in an image	
Maximum: maximal intensity values in an image	
Skewness: asymmetry of the grey-level distribution in the histogram.	$\frac{\frac{1}{E}\sum_i(HISTO(i)-\overline{HISTO})^3}{(\sqrt{\frac{1}{E}\sum_i(HISTO(i)-\overline{HISTO})^2})^3}$
Kurtosis: shape of the grey-level distribution	$\frac{\frac{1}{E}\sum_i(HISTO(i)-\overline{HISTO})^4}{(\frac{1}{E}\sum_i(HISTO(i)-\overline{HISTO})^2)^2}$
Entropy: randomness of the distribution	$-\sum_i p(i)\cdot\log_{10}(p(i)+\varepsilon)$
Energy: uniformity of the distribution	$\sum_i p(i)^2$
Sphericity: how spherical a volume of interest is.	$\frac{\pi^{1/3}\cdot(6V)^{2/3}}{A}$
Compacity: how compact the Volume of Interest is.	$\frac{A^{3/2}}{V}$
Volume (mL and voxels)	$\sum_i V_i$
Grey-level Co-occurrence Matrix (GLCM) features[2]	
GLCM_homogeneity: homogeneity of grey-level voxel pairs	Average over 13 directions $(\sum_i\sum_j\frac{GLCM(i,j)}{1+\lvert i-j\rvert})$
GLCM_Energy: uniformity of grey-level voxel pairs	Average over 13 directions $(\sum_i\sum_j GLCM(i,j)^2)$
GLCM_Contrast: local variations in the GLCM	Average over 13 directions $(\sum_i\sum_j(i,j)^2\cdot GLCM(i,j))$
GLCM_Correlation: linear dependency of grey-levels in GLCM	Average over 13 directions $(\sum_i\sum_j\frac{(i-\mu_i)\cdot(j-\mu_j)\cdot GLCM(i,j)^2}{\sigma_i\sigma_j})$
GLCM_Entropy: randomness of grey-level voxel pairs	Average over 13 directions $(-\sum_i\sum_j GLCM(i,j)\cdot\log_{10}(GLCM(i,j)+\varepsilon))$
GLCM_Dissimilarity: variation of grey-level voxel pairs	$\sum_i\sum_j\lvert i-j\rvert\cdot GLCM(i,j)$
Grey-level Run-Length Matrix (GLRLM) features*	
GLRLM_Short-Run Emphasis (GLRLM_SRE): distribution of the short homogeneous runs in an image	Average over 13 directions $(\frac{1}{H}(\sum_i\sum_j\frac{GLRLM(i,j)}{j^2}))$

GLRLM_Long-Run Emphasis (GLRLM_LRE): distribution of the long homogeneous runs in an image	Average over 13 directions $(\frac{1}{H}(\sum_i\sum_j GLRLM(i,j)\cdot j^2))$
GLRLM_Low Grey-level Run Emphasis (GLRLM_LGRE): distribution of the low grey-level runs	Average over 13 directions $(\frac{1}{H}(\sum_i\sum_j \frac{GLRLM(i,j)}{i^2}))$
GLRLM_High Grey-level Run Emphasis (GLRLM_HGRE): distribution of the high grey-level runs	Average over 13 directions $(\frac{1}{H}(\sum_i\sum_j GLRLM(i,j)\cdot i^2))$
GLRLM_Short-run Low Grey-level Emphasis (GLRLM_SRLGE): distribution of the short homogeneous runs with low grey-levels	Average over 13 directions $(\frac{1}{H}(\sum_i\sum_j \frac{GLRLM(i,j)}{i^2 j^2}))$
GLRLM_Short-run High Grey-level Emphasis (GLRLM_SRHGE): distribution of the short homogeneous runs with low high-levels	Average over 13 directions $(\frac{1}{H}(\sum_i\sum_j \frac{GLRLM(i,j)\cdot i^2}{j^2}))$
GLRLM_Long-run Low Grey-level Emphasis (GLRLM_LRLGE): distribution of the long homogeneous runs with low grey-levels	Average over 13 directions $(\frac{1}{H}(\sum_i\sum_j \frac{GLRLM(i,j)\cdot j^2}{i^2}))$
GLRLM_Long-run Low Grey-level Emphasis (GLRLM_LRHGE): distribution of the long homogeneous runs with high grey-levels	Average over 13 directions $(\frac{1}{H}(\sum_i\sum_j GLRLM(i,j)\cdot i^2\cdot j^2))$
GLRLM_Grey-level Non-uniformity for Run (GLRLM_GLNUr): non-uniformity of the grey-levels	Average over 13 directions $(\frac{1}{H}\sum_i(\sum_j GLRLM(i,j))^2)$
GLRLM_Run Length Non-uniformity (GLRLM_RLNU): length of the homogeneous runs	Average over 13 directions $(\frac{1}{H}\sum_j(\sum_i GLRLM(i,j))^2)$
GLRLM_Run Percentage (GLRLM_RP): homogeneity of the homogeneous runs	Average over 13 directions $(\frac{H}{\sum_i\sum_j(j\cdot GLRLM(i,j))})$
Neighborhood Grey-level Difference Matrix (NGLDM) features[3]	
NGLDM_Coarseness: level of spatial rate of change in intensity	$\frac{1}{\sum_i NGLDM(i,1)\cdot NGLDM(i,2)}$
NGLDM_Contrast: intensity difference between neighboring regions	$\left[\sum_i\sum_j NGLDM(i,1)\cdot NGLDM(j,1)\cdot(i-j)^2\right]\cdot\frac{\sum_i NGLDM(i,2)}{E\cdot G\cdot(G-1)}$
NGLDM_Busyness: spatial frequency of changes in intensity	$\frac{\sum_i NGLDM(i,1)\cdot NGLDM(i,2)}{\sum_i\sum_j\lvert(i\cdot NGLDM(i,1)-j\cdot NGLDM(j,1)\rvert}$ with NGLDM(i,1)≠1, NGLDM(j, 1)≠0
Grey-level Zone Length Matrix (GLZLM) features†[4]	
GLZLM_Short-zone Emphasis (GLZLM_SZE): distribution of the short homogeneous zones in the image	$\frac{1}{H}(\sum_i\sum_j\frac{GLZLM(i,j)}{j^2})$
GLZLM_Long-zone Emphasis (GLZLM_LZE): distribution of the long homogeneous zones in the image	$\frac{1}{H}(\sum_i\sum_j GLZLM(i,j)\cdot j^2)$
GLZLM_Low Grey-level Zone Emphasis (GLZLM_LGZE): distribution of the low grey-level zones	$\frac{1}{H}(\sum_i\sum_j\frac{GLZLM(i,j)}{i^2})$

GLZLM_High Grey-level Zone Emphasis (GLZLM_LGZE): distribution of the high grey-level zones	$\frac{1}{H}(\sum_i \sum_j GLZLM(i,j) \cdot i^2)$
GLZLM_Short-zone Low Grey-level Emphasis (GLZLM_SZLGE): distribution of the short homogeneous zones with low grey-levels	$\frac{1}{H}(\sum_i \sum_j \frac{GLZLM(i,j)}{i^2 \cdot j^2})$
GLZLM_Short-zone High Grey-level Emphasis (GLZLM_SZHGE) : distribution of the long homogeneous zones with low grey-levels	$\frac{1}{H}(\sum_i \sum_j \frac{GLZLM(i,j) \cdot i^2}{j^2})$
GLZLM_Long-zone Low Grey-level Emphasis (GLZLM_LZLGE): distribution of the long homogeneous zones with low grey-levels	$\frac{1}{H}(\sum_i \sum_j \frac{GLZLM(i,j) \cdot j^2}{i^2})$
GLZLM_Long-zone High Grey-level Emphasis (GLZLM_LZHGE) : distribution of the long homogeneous zones with high grey-levels	$\frac{1}{H}(\sum_i \sum_j GLZLM(i,j) \cdot i^2 \cdot j^2)$
GLZLM_Grey-level Non-uniformity for Zone (GLZLM_GLNUz) : non-uniformity of the grey-levels	$\frac{1}{H}\sum_i \left(\sum_j GLZLM(i,j))\right)^2$
GLZLM_Zone Length Non-uniformity (GLZLM_ZLNU): length of the homogeneous zones	$\frac{1}{H}\sum_j \left(\sum_i GLZLM(i,j))\right)^2$
GLZLM_Zone Percentage (GLZLM_ZP): homogeneity of the homogeneous zones	$\frac{H}{\sum_i \sum_j (j \cdot GLZLM(i,j))}$

*H, the number of homogeneous runs in ROI;

†H, the number of homogeneous zones in ROI;

V, volume of region of interest (ROI) ; A, surface of ROI; V_i, volume of voxel i of ROI; E, the total number of voxels in ROI; HISTO(i), the number of voxels with intensity i; $\overline{HISTO}$, the average of grey-levels in the histogram; $P(i)$, probability of occurrence of voxels with intensity I; ε = 2e-16;

References

1. Nioche C, Orlhac F, Boughdad S, et al. Lifex: A freeware for radiomic feature calculation in multimodality imaging to accelerate advances in the characterization of tumor heterogeneity. *Cancer Research* 2018:canres.0125.2018.
2. Haralick RM, Shanmugam K. Textural features for image classification. *IEEE Transactions on systems, man, and cybernetics* 1973:610-621.
3. Amadasun M, King R. Textural features corresponding to textural properties. *IEEE Transactions on Systems, Man, and Cybernetics* 1989;19:1264-1274.
4. Xu D-H, Kurani AS, Furst JD, et al. Run-length encoding for volumetric texture. *Heart* 2004;27:452-458.
