# Supplementary material for: Magnetic resonance imaging features of tumor and lymph node to predict clinical outcome in node-positive cervical cancer: a retrospective analysis
Source: Radiat Oncol. 2020 Apr 20;15:86. doi: 10.1186/s13014-020-01502-w (PMC7171757; doi:10.1186/s13014-020-01502-w)
Supplement: Supplementary file 2 — Additional file 2. Univariate analysis of potential prognostic factors for overall survival in the validation dataset. [file 13014_2020_1502_MOESM2_ESM.pdf]

Supplementary Table 3. Univariate analysis of potential prognostic factors for overall survival in the validation dataset

Variables		n	5-year OS (%)	*P* value
Imaging feature-based survival risk score[1]	<2.6	17	80.0	0.569
	≥2.6	15	93.8	
Age	<50	15	92.3	0.592
	≥50	17	100.0	
Pathology	SCC	30	91.5	0.882
	Non-SCC	2	100.0	
FIGO stage[2]	IIB	25	86.1	0.729
	IIIA, IIIB	7	100.0	
Extent of nodal involvement	Pelvic only	25	88.4	0.779
	Pelvic+para-aortic	7	83.3	
Primary tumor size (mm)	<50	16	90.9	1.000
	≥50	16	83.9	

Abbreviations: RC = regional control; HR = hazard ratio; CI = confidence interval; SCC = squamous cell carcinoma

[1] Imaging features of involved lymph nodes

[2] The 2009 International Federation of Gynecology and Obstetrics (FIGO) staging system
