# Supplementary figures and images for: Magnetic resonance imaging features of tumor and lymph node to predict clinical outcome in node-positive cervical cancer: a retrospective analysis
Source: Radiat Oncol. 2020 Apr 20;15:86. doi: 10.1186/s13014-020-01502-w (PMC7171757; doi:10.1186/s13014-020-01502-w)

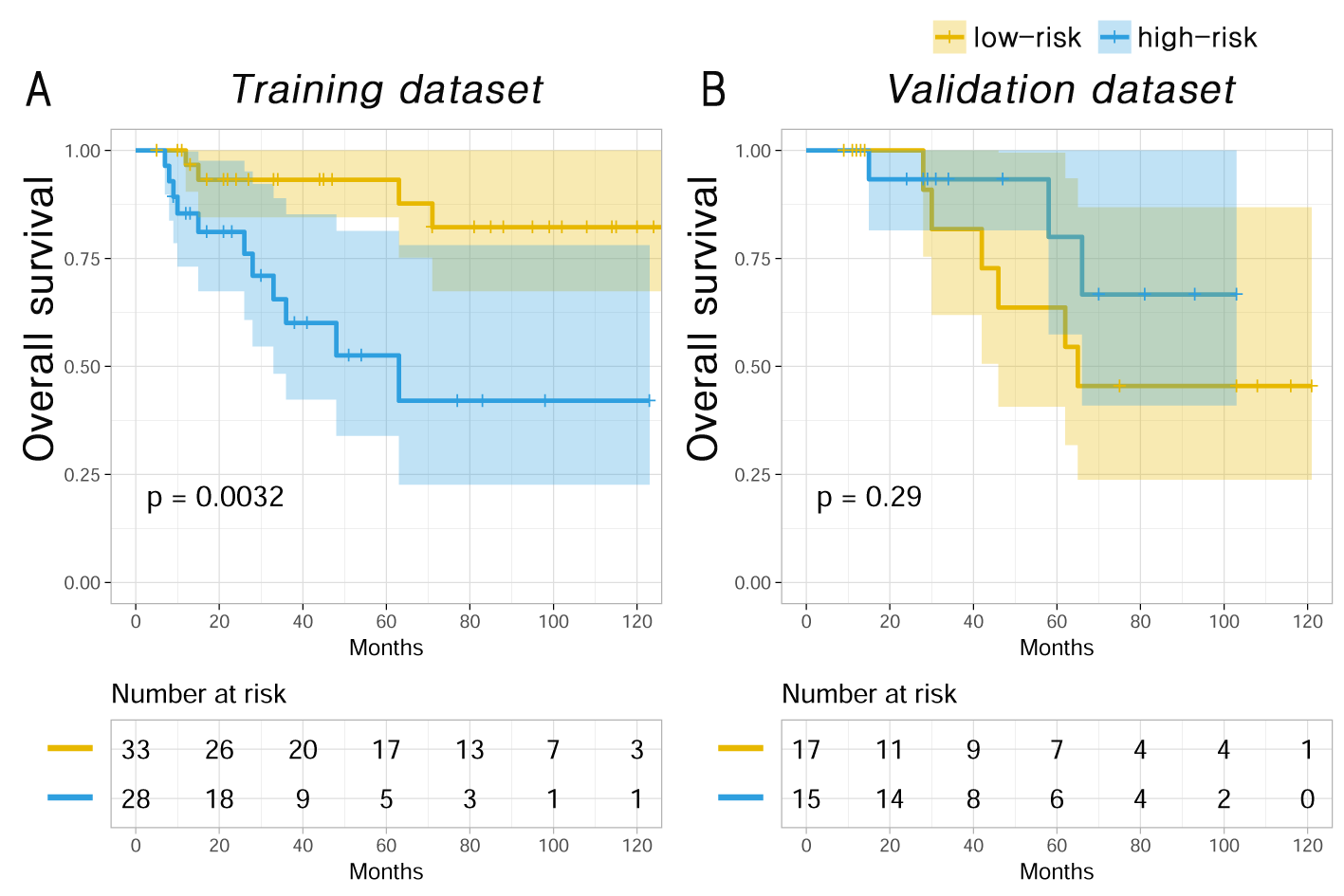

Supplement: Supplementary file 3 — Additional file 3. Kaplan–Meier curves of overall survival for patients in the training dataset (A) and validation dataset (B) stratified into low- and a high-risk groups. [file 13014_2020_1502_MOESM3_ESM.tif]
